# High-Level Production of a Recombinant Protein in *Nicotiana benthamiana* Leaves Through Transient Expression Using a Double Terminator

**DOI:** 10.3390/ijms252111573

**Published:** 2024-10-28

**Authors:** Jihyea Lee, Kyeong-Ryeol Lee, Nan-Sun Kim, Juho Lee, Seon-Kyeong Lee, Sichul Lee

**Affiliations:** Department of Agricultural Biotechnology, National Institute of Agricultural Sciences, Rural Development Administration, Jeonju 54875, Republic of Korea; liah1026@korea.kr (J.L.); nskims@korea.kr (N.-S.K.); jhlee0102@korea.kr (J.L.); lsk220@korea.kr (S.-K.L.); sciron1015@korea.kr (S.L.)

**Keywords:** terminator, polyadenylation, transient expression, *Nicotiana benthamiana*

## Abstract

Various bio-based recombinant proteins have been produced for industrial, medical, and research purposes. Plants are potential platforms for recombinant protein production because of several advantages. Therefore, establishing a system with high target gene expression to compensate for the low protein yield of plant systems is crucial. In particular, selecting and combining strong terminators is essential because the expression of target genes can be substantially enhanced. Here, we aimed to quantify the enhancement in the fluorescence intensity of the turbo green fluorescence protein (tGFP) caused by the best double-terminator combinations compared to that of the control vector using agroinfiltration in *Nicotiana benthamiana* leaves. tGFP fluorescence increased by 4.1-fold in leaf samples infiltrated with a vector containing a double terminator and markedly increased by a maximum of 23.7-fold when co-infiltrated with the geminiviral vector and P19 compared to that in constructs containing an octopine synthase terminator. Polyadenylation site analysis in leaf tissues expressing single or dual terminators showed that the first terminator influenced the polyadenylation site determination of the second terminator, resulting in different polyadenylation sites compared with when the terminator is located first. The combination of the high-expression terminators and geminiviral vectors can increase the production of target proteins.

## 1. Introduction

Numerous useful recombinant proteins, such as antigens, antibodies, and enzymes, are produced using expression systems in *Escherichia coli*, yeast, insect cells, and mammalian cells for industrial, medical, and research purposes [1]. Recently, recombinant protein production systems using plants have emerged owing to their various advantages, such as low potential for contamination with endotoxins and commensal pathogens, ease of scale-up, and low upstream production costs [2,3]. However, their limitations include a low recombinant protein yield and high purification costs [4,5,6].

Several strategies have been developed to increase the efficiency of plant-based recombinant protein production. First, a viral expression system is used. Viral vectors can be engineered to eliminate virulence and deliver target genes into the cell for expression, as viruses infiltrate host cells and replicate using the replication machinery of the host cell [7]. For example, the magnICON system uses the RNA replication systems of tobacco mosaic virus (TMV) and potato virus X (PVX), and the in-plant activation (INPACT) expression system uses the DNA replication system of geminivirus, a DNA virus [7,8]. Second, co-expression of virus-induced gene silencing suppressors, such as P19 from tomato bushy stunt virus (TBSV) and P25 from PVX, is also used [9]. Post-transcriptional gene silencing (PTGS) prevents externally introduced genes from being transcribed [10,11], and virus-derived proteins such as P19 and P25 can increase gene expression by acting as suppressors during this process [11,12]. Third, codon optimization of the target gene matches the codon usage in the host [13,14]. Fourth, using *Agrobacterium tumefaciens* strains can increase the target protein yield. However, the efficiency of transgene expression by various *Agrobacterium* strains varies depending on the plant species and expression system; for example, the GV3101 strain shows higher expression efficiency than the LBA4404 strain in *Nicotiana benthamiana* [15]. Furthermore, gene expression is approximately two times higher when using the *A. tumefaciens* strain EHA105 than when using GV3101 and LBA4404 [16]. Fifth, host plants with high expression of recombinant proteins, such as *N. benthamiana* and rice (*Oryza sativa* L.), should be selected [17]. Sixth, stronger promoters such as cauliflower mosaic virus (CaMV) 35S and heat shock protein can be used to further increase gene expression [18]. Seventh, the use of a 5′-untranslated region (UTR) can increase translation efficiency [19].

In addition, selecting and combining terminators can facilitate the upregulation of target gene expression. During gene transcription, various protein complexes recognize and bind various motifs located in the terminator in a series of 3′-end processing steps that involve cleaving pre-mRNA and forming a polyadenylic acid (poly(A)) tail at the 3′-end. Although these motif sequences appear similar within mammal and yeast terminators [20], plant terminators have slightly different motif sequences. Plant terminators have three main regulatory elements—a far upstream element (FUE), a near upstream element (NUE), and a cleavage element (CE). The NUE is an A-rich region with a sequence similar to the AAUAAA motif, known as the polyadenylation (PA) signal in mammalian terminators, located 10–30 nucleotides upstream of the cleavage site. The FUE is a UG-rich region that is located 50–160 nucleotides upstream of the cleavage site, and CE is an A/U-rich region of approximately 20 nucleotides before and after the cleavage site [21]. During 3′-end processing, protein complexes recognize and bind the FUE and NUE motifs, the CE is cleaved at the cleavage site by forming a hairpin structure, and a poly(A) tail is formed from the cleavage site [21,22]. Read-through transcription due to poorly functioning terminators results in abnormally elongated mRNA that lacks a poly(A) tail, which is produced as double-stranded RNA (dsRNA) by RNA-dependent RNA polymerase 6 (RDR6). dsRNA is then cleaved by DICER-LIKE 2 and four RNase III enzymes into 21–22-nucleotide small interfering RNAs (siRNAs) [23]. The siRNA forms an RNA-induced silencing complex with Argonaute proteins, which cleaves the complementary target mRNA, and dsRNA is generated by RDR6 from the cleaved siRNA, thereby increasing the effectiveness of PTGS [24,25]. Therefore, using strong terminators can enhance mRNA stability by increasing the efficiency of PA and decreasing PTGS, which results in elevated gene expression [17,18,25,26]. Combinations of different terminators further increase the efficiency of PA, substantially enhancing the expression of target genes [17,18,27,28,29].

Geminivirus-based deconstructed vectors with a DNA replicating system have been used to increase gene expression. Members of the *Begomovirus* genus, belonging to geminiviruses, have a unique replication method that uses an intergenic region (IR) and replication-related proteins (C1–C4), including replication initiation proteins (Rep, C1) that recognize IRs and are involved in replication [30,31]. IRs have an origin of replication (ORI), which forms a stem-loop structure. The Rep protein recognizes the ORI in the IRs and forms episomal replicons [32]. Repeating the rolling circle replication results in the formation of episomal replicons with high copy numbers, which eventually synthesize multiple viral proteins [32,33,34]. In addition, proteins are synthesized through C2–C4 OFRs that function as replication assistants. Therefore, deconstructed viral vectors created by combining these viral elements can be used to increase target protein production.

Until recently, studies have reported enhanced expression of target genes via agroinfiltration with plant expression vectors carrying reporter genes encoding green fluorescence protein (GFP), yellow fluorescence protein, and β-glucuronidase (GUS) with double terminators in plants [17,28,35,36]. In this study, to determine the extent to which the expression of the target gene was enhanced by 13 combinations of double terminators not reported in previous studies and to determine the best combination, we selected two weak single terminators, namely CaMV 35S (35ST) and *Solanum lycopersicum* RbcS3C (RbcST), and three strong single terminators, including *N. tabacum* Extensin (ExtT), *N. benthamiana Actin3* (Act3T), and *S. tuberosum* protease inhibitor II (PinIIT). Furthermore, we examined whether the combined use of double terminators and deconstructed geminiviral vectors based on the tomato yellow leaf curl virus (TYLCV) belonging to the genus *Begomovirus* [32], which was created in a previous study [37], can enhance turbo GFP (tGFP) expression compared to the single use of a deconstructed TYLCV-based vector. Additionally, we analyzed how the use of double terminators and TYLCV-based vectors increases the fluorescence intensity and protein quantity of tGFP. The plant-based system for enhanced expression of target proteins, consisting of a double terminator and a deconstructed TYLCV-based vector, used in this study could contribute to the commercial-scale production of target proteins, including vaccines and pharmaceuticals.

## 2. Results

### 2.1. Double Terminators Increase tGFP Expression

**Figure 1:**
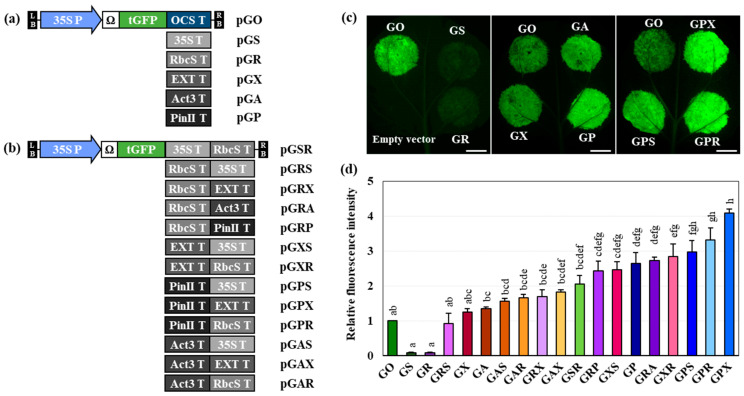
Relative fluorescence intensities of turbo green fluorescence protein (tGFP) in hydroponically grown *Nicotiana benthamiana* leaf samples agroinfiltrated with the vector constructs for tGFP expression with single or double terminators. (**a**,**b**) tGFP expression vector constructs containing (**a**) single or (**b**) double terminators. A vector with a 35S dual promoter and a tobacco mosaic virus 5′-untranslated region Ω was used to express tGFP. (**c**) tGFP fluorescence in leaves agroinfiltrated with vector constructs with single or double terminators at 3 days post infiltration (DPI). Scale bar: 1 cm. (**d**) Relative tGFP fluorescence intensity of the leaf samples was calculated using a GO (tGFP:OCST) value of 1. Statistical analysis was performed using a one-way analysis of variance (ANOVA), followed by Tukey’s test (*p* < 0.05).

tGFP was transiently expressed using agroinfiltration in the greenhouse and hydroponically grown *N. benthamiana* leaves to determine under which condition *N. benthamiana* had higher tGFP content. Binary vectors for tGFP expression containing five single terminators and 13 double terminators were constructed (Figure 1a,b), and the effects of these terminators on the enhancement of *tGFP* transgene expression were compared. Transient expression in greenhouse-grown plants resulted in a low fluorescence intensity for GS (tGFP:35ST) and GR (tGFP:RbcST) (Figure S1a). GP (tGFP:PinIIT) showed the highest tGFP expression among the single terminators, which was approximately 2.6-fold higher than that of GO (tGFP:OCST), which was used as the control. The fluorescence intensity using double terminators containing PinIIT was higher than that of other constructs, and GPR (tGFP:PinIIT–RbcST) showed the highest fluorescence intensity (approximately 7.3-fold higher than that of GO) among the constructs containing double terminators (Figure S1b). Fluorescence quantification of GS and GR was not performed because fluorescence was barely visible. Similarly, transient expression in hydroponically grown plants showed low fluorescence intensity for GS and GR (Figure 1c), as low as 10% of that of GO (Figure 1d). PinIIT was the strongest in terms of fluorescence intensity using a single terminator, followed by Act3T; ExtT was comparable to Act3T (Figure 1c,d).

The relative fluorescence intensity and quantity of transiently expressed tGFP in hydroponically grown *N. benthamiana* were significantly higher than those in greenhouse-grown *N. benthamiana* (*p* < 0.05) (Figure 1 and Figure S1b,c). This result is likely because compared to the conditions for soil-grown plants in a greenhouse, hydroponics offer more uniform environmental conditions and a constant supply of nutrient media, which is conducive to efficient protein production. Notably, the tGFP fluorescence intensities of GS and GR were undetectable in greenhouse-grown *N. benthamiana* (Figure S1a) but were detected in small amounts in hydroponically grown *N. benthamiana* at 10% relative to those of GO (Figure 1c and Figure S1c). Although the tGFP fluorescence intensity of the other constructs did not increase at the same rate, the overall increase was greater in hydroponically grown *N. benthamiana* than in greenhouse-grown *N. benthamiana*, and the difference was statistically significant (Figure S1c). Accordingly, all subsequent experiments were performed using hydroponically grown *N. benthamiana*.

The leaf samples infiltrated with GX, GA, or GP exhibited higher fluorescence intensities than the GO-infiltrated leaf sample, with the fluorescence intensity of the GP-infiltrated leaf sample being the highest among the constructs with a single terminator, at approximately 2.6-fold that of the GO-infiltrated leaf sample (Figure 1d). Interestingly, GRS (tGFP:RbcST–35ST) induced the lowest fluorescence intensity among the constructs with double terminators (Figure 1d). However, the fluorescence intensity of the GRS-infiltrated leaf sample was 92% that of the GO-infiltrated leaf sample, which was 9.8-fold higher than that of GS (Figure 1d). GSR (tGFP:35ST–RbcST) exhibited a 24.4-fold higher fluorescence intensity than GS, which was 2.1-fold higher than that of GO (Figure 1d). The fluorescence intensities of GPS (tGFP:PinIIT–35ST), GPR (tGFP:PinIIT–RbcST), and GPX (tGFP:PinIIT–ExtT), which are constructs with double terminators wherein another terminator was added to the 3′-end of PinIIT, were the highest—3.0–4.1-fold higher than those of GO and approximately 1.2–1.7-fold higher than those of GP (Figure 1d). The fluorescence intensities of GPR and GPS with PinIIT significantly increased by approximately 35-fold compared to those of GR and GS, which exhibited significantly low fluorescence intensities. They had the second- and third-highest fluorescence intensities, respectively, among the 13 constructs with double-terminator combinations. Furthermore, the fluorescence intensity of the GRP (tGFP:RbcST–PinIIT)-infiltrated leaf sample was 2.4-fold higher than that of the GO-infiltrated leaf sample but only 68% higher than that of the GPR-infiltrated leaf sample (Figure 1d).

### 2.2. Fluorescence Intensity of tGFP Is Significantly Stronger When It Is Co-Expressed with TYLCV Rep Than Without TYLCV Rep

**Figure 2:**
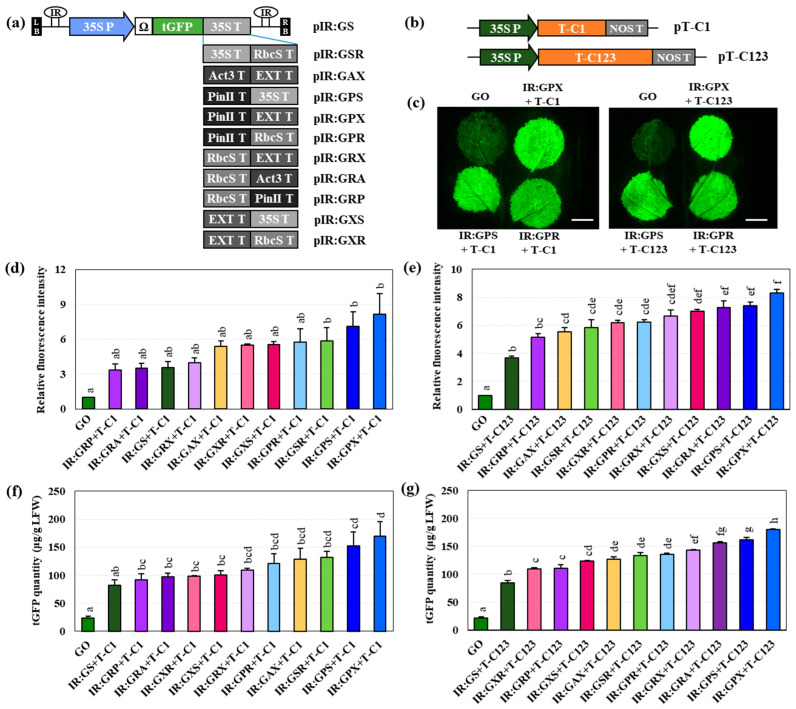
Relative tGFP fluorescence intensities of *N. benthamiana* leaf samples co-infiltrated with pT–C1 or pT–C123 and the replicating vector constructs for tGFP expression containing double terminators. (**a**) Replicating vector constructs with cauliflower mosaic virus 35S or double terminator containing TYLCV IRs at both ends to form an episomal replicon. (**b**) Vector constructs (pT-C1 and pT-C123) expressing the Rep (T-C1) and replication-related genes (T-C123) from TYLCV, respectively. (**c**) tGFP fluorescence in leaves co-infiltrated with pT–C1 or pT–C123 and the replicating vector constructs containing double terminators at 3 DPI. Scale bar: 1 cm. (**d**,**e**) Relative tGFP fluorescence intensity of the leaf samples co-infiltrated with (**d**) T–C1 or (**e**) T–C123 and the replicating vector construct was calculated using a GO value of 1. (**f**,**g**) tGFP quantity of the leaf sample co-infiltrated with (**f**) T–C1 or (**g**) T–C123 and replicating vector constructs containing double terminators at 3 DPI. Statistical analysis was performed using a one-way ANOVA followed by Tukey’s test (*p* < 0.05).

The top 10 constructs with double-terminator combinations were selected by comparing the fluorescence intensities of the 18 constructs with single or double terminators for tGFP expression. Ten constructs of TYLCV IR were added to both ends of the *tGFP* transcription unit (Figure 2a) and co-infiltrated with TYLCV Rep (T–C1 and T–C123) to confirm the enhancement of tGFP fluorescence intensity (Figure 2b). Agroinfiltration of constructs with only TYLCV IR attached to both ends of the *tGFP* transcription unit without the Rep protein did not increase tGFP expression but instead decreased it (Figure S2). This finding is likely because IR, which is a replication site and bidirectional promoter, only acts as a promoter in the absence of Rep, thus interfering with *tGFP* transcription. Interestingly, the fluorescence intensity of the GS-infiltrated leaf sample was low, but that of the IR:GS-infiltrated leaf sample was significantly enhanced to 77% of that of the GO-infiltrated leaf sample, which is a 13.5-fold increase compared to that in the GS-infiltrated leaf sample.

tGFP and TYLCV Rep co-expression resulted in an increase in tGFP fluorescence intensity by approximately 2-fold compared to that achieved with tGFP expression alone (Figure 1 and Figure 2). In particular, GPX co-expressed with TYLCV Rep exhibited the highest fluorescence intensity among the 10 constructs (Figure 2c–e). GPS and GPX co-expression with T–C123 resulted in higher tGFP fluorescence intensities than with T–C1, increasing by 7.4- and 8.3-folds, respectively, compared to that of GO (Figure 2d,e). Notably, the tGFP fluorescence intensities of GS- and IR:GS-infiltrated leaf samples were approximately 10% and 77%, respectively, compared to those of the GO-infiltrated leaf samples (Figure 1d and Figure S2b). In contrast, IR:GS and TYLCV Rep co-expression increased the fluorescence intensity up to 3.7-fold compared to that of the GO-infiltrated leaf sample (Figure 2d,e).

The ranking of tGFP content was slightly different from the ranking of relative fluorescence intensity. For co-expression of T–C1 or T–C123, the top three rankings for relative fluorescence intensity and tGFP content were similar to each other but otherwise differed slightly (Figure 2d–g). tGFP levels ranged from 82.7 to 169.8 μg/g LFW with T–C1 co-expression (Figure 2f) and 85.1–180.4 μg/g LFW with T–C123 co-expression (Figure 2g). The top six constructs that showed the highest tGFP content upon co-expression with T–C1 (IR:GAX, IR:GSR, IR:GPR, IR:GRX, IR:GPS, and IR:GPX) or T–C123 (IR:GRX, IR:GSR, IR:GRA, IR:GPR, IR:GPS, and IR:GPX) were used for further investigation (Figure 2f,g).

### 2.3. Transient tGFP Expression Was Enhanced with P19 Co-Expression

**Figure 3:**
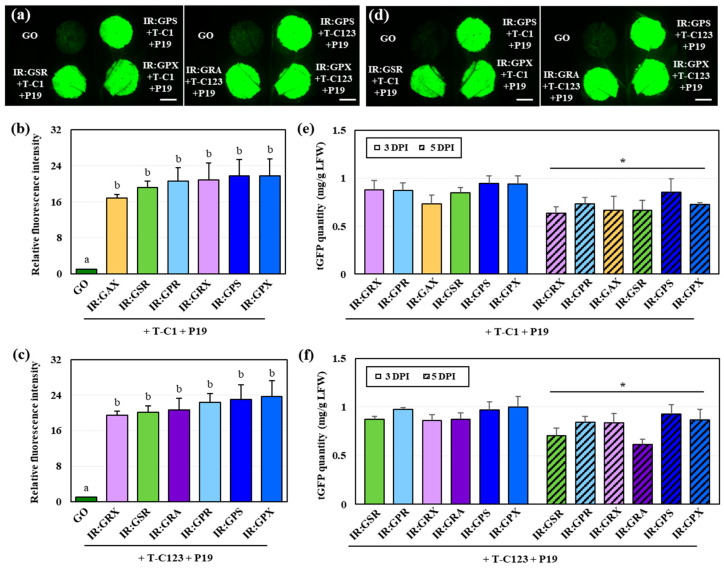
Relative tGFP fluorescence intensities and quantities in *N. benthamiana* leaf samples co-infiltrated with the vector constructs for tGFP expression containing double terminators, pT–C123, and P19 at 3 and 5 DPI. (**a**,**d**) tGFP fluorescence of each construct upon transient expression at (**a**) 3 and (**d**) 5 DPI. Scale bar: 1 cm. (**b**,**c**) The relative tGFP fluorescence intensity of leaf samples co-infiltrated with the replicating vector construct, P19, and (**b**) T–C1 or (**c**) T–C123. The values were statistically analyzed using a one-way ANOVA followed by Tukey’s test (*p* < 0.05). (**e**,**f**) tGFP quantity of leaf samples co-infiltrated with the replicating vector construct, P19, and (**e**) T–C1, or (**f**) T–C123 at 3 and 5 DPI. The values were statistically analyzed using Wilcoxon’s signed-rank test (* *p* < 0.05).

The selected constructs were used to investigate the quantity of tGFP that accumulates upon additional co-expression with P19. The fluorescence intensity of all leaf samples co-infiltrated with P19 and T–C1 or T–C123 was much higher than that of the GO-infiltrated leaf samples at 3 DPI (Figure 3a). Furthermore, co-infiltration with P19 remarkably (2.7–5.3-fold) increased the relative fluorescence intensity compared to that without P19 (Figure 2d,e and Figure 3b,c). Compared to that of GO, the fluorescence intensity of samples co-infiltrated with T–C1 and P19 increased by 16.9–21.7-fold, and that of samples co-infiltrated with T–C123 and P19 increased by 19.5–23.7-fold, indicating a slightly greater increase with T–C123 co-infiltration than with T–C1 co-infiltration (Figure 3b,c). The tGFP content and fluorescence intensity of GPS and GPX co-infiltrated with T–C1+P19 or T–C123+P19 were the highest among the six constructs, respectively, and there was a difference in the relative fluorescence intensity of the six constructs. However, there were no statistically significant differences among the six constructs for both co-expression cases (Figure 3b,c). Regarding co-expression of both T–C1 and T–C123, the fluorescence intensity was still strong at 5 DPI (Figure 3d), similar to that at 3 DPI; however, the fluorescence intensity of the 5 DPI samples was significantly lower than that of the 3 DPI samples (*p* < 0.05) (Figure 3e,f). These results show that co-expression of P19, a gene silencing suppressor, can remarkably increase the target protein expression when using TYLCV-based vectors and that there is a limit to increasing the target gene expression without the use of P19.

### 2.4. tGFP Quantity Accumulates in Sodium Dodecyl-Sulfate Polyacrylamide Gel Electrophoresis and Enzyme-Linked Immunosorbent Assay

Total soluble proteins (TSPs) were extracted from the samples, and sodium dodecyl-sulfate polyacrylamide gel electrophoresis (SDS-PAGE) and enzyme-linked immunosorbent assay (ELISA) were performed using the extracted TSPs. This analysis was conducted to determine the tGFP content and TSP ratio in the infiltrated samples of the GPS and GPX constructs, which had the highest expression based on the comparative analysis of the tGFP fluorescence intensity of each construct (Figure 1, Figure 2 and Figure 3).

**Figure 4:**
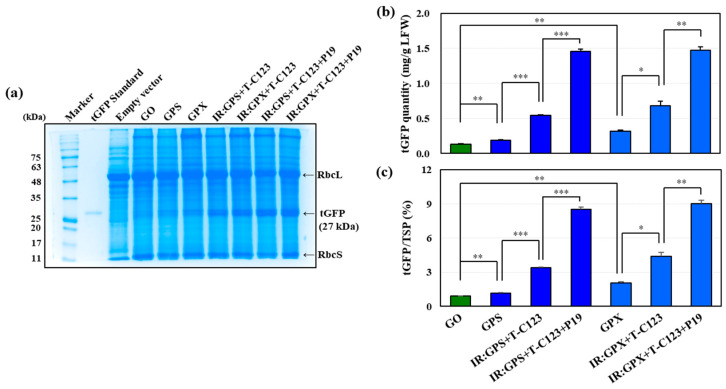
Recombinant tGFP accumulation in the sample agroinfiltrated with each diverse construct. Total soluble protein (TSP) was extracted from the infiltrated samples and subjected to sodium dodecyl-sulfate polyacrylamide gel electrophoresis (SDS-PAGE) and enzyme-linked immunosorbent assay (ELISA). (**a**) SDS-PAGE with Coomassie Brilliant Blue staining. As a positive control, 100 ng of tGFP standard was loaded, and TSP from a leaf sample infiltrated with a level M empty vector was used as a negative control. TSP (25 μg) from all leaf samples was loaded. RbcL and RbcS are ribulose-1,5-bisphosphate carboxylase/oxidase large and small subunits, respectively (**b**) The tGFP quantity of leaf samples agroinfiltrated with the diverse vector constructs was confirmed using indirect ELISA. (**c**) The proportion of tGFP in TSP was calculated using the TSP concentration and ELISA results. Statistical analysis was performed using a one-way ANOVA (* *p* < 0.05; ** *p* < 0.01; *** *p* < 0.001).

A tGFP band of 27 kDa in size was identified, and the tGFP band intensity increased with the use of T–C123 and P19 in the GPS and GPX constructs (Figure 4a). The amount of tGFP in the GO sample was 0.13 mg/g leaf fresh weight (LFW), whereas GPS and GPX were 0.19 and 0.32 mg/g LFW, respectively (Figure 4b). This finding indicates that a higher amount of tGFP can accumulate with the double terminator than with the single terminator, which is consistent with the comparison of fluorescence intensities (Figure 1 and Figure S1b). Furthermore, the tGFP content of the leaf sample co-infiltrated with IR:GPX + T–C123 was 0.69 mg/g LFW, which was approximately 2.2-fold higher than that of the GPX infiltrated sample, which was 0.32 mg/g LFW (Figure 4b). Co-infiltration with P19 (IR:GPX + T–C123 + P19) resulted in 1.47 mg/g LFW of tGFP, an increase of approximately 2.1-fold compared to that in the absence of P19 (IR:GPX + T–C123) (Figure 4b). This result was equivalent to 2% TSP for GPX, increasing to 3.4% TSP for IR:GPX + T–C123, and significantly increasing to 9% TSP for IR:GPX + T–C123 + P19 (Figure 4c). The tGFP quantities of GPS and IR:GPS + T–C123 were 60% and 80% of those of GPX and IR:GPX + T–C123, respectively, but that of IR:GPS + T–C123 + P19 accounted for 8.5% of the TSP, which was comparable to that of IR:GPX + T–C123 + P19 (Figure 4b,c). The statistical significance between each step showed that the use of double terminators, the use of TYLCV-based vectors, and the co-expression of P19 were all synergistic.

### 2.5. PA Site Changes When It Is Located in the Second Terminator

**Figure 5:**
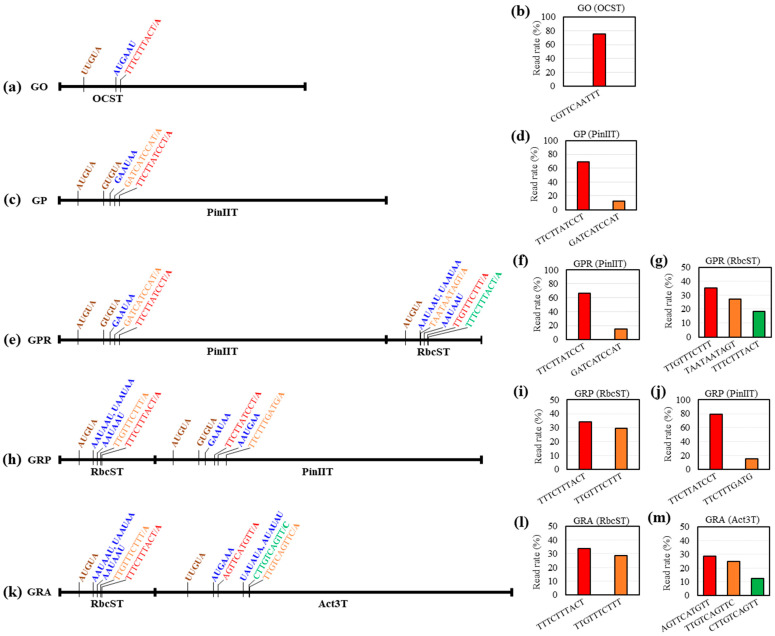
Analysis of polyadenylation (PA) site and putative PA signal motif of each construct with a single or double terminator. (**a**,**c**,**e**,**h**,**k**) Diagram of each terminator region in a single or double terminator construct used to analyze the PA site. Brown and blue bold letters are putative far upstream element (FUE) and near upstream element (NUE) motifs, respectively. Red, orange, and green letters are the top three PA sites (bold), and the 10 bp sequence upstream of the PA site of each terminator is >10% of the read rate in the analysis results. (**b**,**d**,**f**,**g**,**i**,**j**,**l**,**m**) The read rate for the top three PA sites of each terminator represents the red, orange, and green bars in the diagram.

Five circularized RT-PCR (cRT-PCR) products from samples infiltrated with GO, GP, GPR, GRA, and GRP constructs were used to identify PA sites (Figure 5 and Table 1). The top three PA sites with a read percentage of ≥ 10% containing a 10 bp sequence preceding the PA site are shown in Figure 5, and all results > 5% are shown in Table 1, along with the corresponding putative FUE and NUE motifs.

One primary PA site (CGTTCAATTT/A, 75.1%) was identified in a GO-infiltrated leaf sample, from which a putative NUE motif (AUGAAU) at −20 nt was identified (Figure 5a,b). This NUE motif corresponded to the top 39 of the top 50 NUE patterns [38] in *Arabidopsis* genes (Table 1), which is consistent with the results of Wang et al. [39]. Furthermore, a putative FUE motif (UUGUA) was also identified at −112 nt from the PA site (Figure 5a).

Two PA sites with a read ratio of > 10% were identified in a GP-infiltrated leaf sample, and a primary PA site (TTCTTATCCT/A) with a read ratio of 69.1% was observed (Figure 5c,d). A putative NUE motif (GAAUAA) at −31 nt was then identified, which corresponded to the top 32 NUE patterns, consistent with the results of Wang et al. [39]. Furthermore, a putative FUE motif (GUGUA) was also identified at −125 nt (Figure 5c and Table 1).

In cRT-PCR products from samples using double terminators, PA sites were identified in both terminators (Figure 5e–g), indicating that the second terminator compensated for the abnormal transcription termination of the first terminator. The major PA site (TTCTTATCCT/A) of PinIIT was 66.8% in GPR, which was consistent with that of GP (Figure 5d–f). However, the top three PA sites (TTGTTTCTTT/A (35.2%), TAATAATAGT/A (27.3%), and TTTCTTTACT/A (18.2%)) were identified in RbcST in GPR (Figure 5e,g). From the PA site with the highest read rate, a putative NUE motif corresponding to the top three of the top nine was identified in the AAUAAUAAU region at −25 nt and the AAUAAU region at −14 nt, and a putative FUE motif (AUGUA) at −68 nt was observed (Figure 5e and Table 1).

In the cRT-PCR product from the GRP-infiltrated leaf samples (Figure 5h–j), the primary PA site (TTCTTATCCT/A) of PinIIT was consistent with that of GP and GPR, but the percentage was 79.6%, which was approximately 10% higher than that of GP and GPR (Figure 5h,j). In contrast, the minor PA site (TTCTTTGATG/A) differed from GATCATCCATA in GP and GPR (Figure 5d,f,j). However, differences were observed in the ranking and proportion of each PA site for RbcST in GRP, in contrast to the GPR results. The highest proportion of PA sites in the RbcST of GPR and GRP were TTGTTTCTTT/A and TTTCTTTACT/A, respectively, whereas TAATAATAGT/A, the second highest proportion of PA sites in the RbcST of GPR, was changed to a minor PA site in the RbcST of GRP, accounting for 8.1% of PA sites (Figure 5g,i). In addition, TTTCTTTACT/A, the third primary PA site in the RbcST of GPR, was the most abundant PA site in the RbcST of GRP (Table 1, Figure 5g,i). Because of the short length of RbcST (279 bp), the PA complex is less likely to be recruited at the relatively frontmost PA site (TAATAATAGT/A) among the multiple PA sites, resulting in a poor PA rate. However, the PA complex is relatively well recruited at that site when RbcST is located behind the double terminator combination, resulting in a high PA rate.

In the cRT-PCR products from GRA-infiltrated leaf samples (Figure 5k–m), multiple PA sites in RbcST were consistent with those of GRP, suggesting that the PA sites are determined differently depending on the location of RbcST when the two terminators are combined. Three major PA sites were identified in Act3T (AGTTCATGTT/A (28.8%), TTGTCAGTTC/A (25.0%), and CTTGTCAGTT/C (12.5%)) (Table 1, Figure 5k,m).

## 3. Discussion

We constructed 13 constructs for *tGFP* expression containing double terminators, comprising five single terminators, to examine whether the double terminator combinations can enhance the number of recombinant proteins in *N. benthamiana*. To increase the DNA vector copy number and thereby enhance the recombinant protein yield, we attached TYLCV IRs to both ends of the *tGFP* transcription unit and used this construct with a DNA-replicating TYLCV-based deconstructed vector, which was developed in a previous study [37], to co-infiltrate *N. benthamiana* leaves. Finally, we co-agroinfiltrated P19 with a tGFP expression construct and TYLCV-based viral vector into *N. benthamiana* to quantify the increase in content and determine the optimal double terminator combination. The findings of this study are as follows: (1) Double terminators led to a significant increase in tGFP fluorescence intensity compared to single terminators. (2) Co-expression of the TYLCV-based viral vector and a *tGFP* expression construct containing a double terminator showed synergistic effects in *N. benthamiana*. (3) Co-expression of the TYLCV-based viral vector and GPX construct with the gene silencing suppressor P19 yielded approximately 1.47 mg tGFP/g LFW, indicating 29% increased content compared to that achieved with the co-expression of the TYLCV-based viral vector, GP construct, and P19 [37]. (4) Analysis of the PA site suggests that the interaction between the first and second terminators within a double terminator construct plays a crucial role in determining PA site usage and overall gene expression levels. These findings indicate that the use of the TYLCV-based viral vector system P19 and the combination of the strong terminators PinIIT and ExtT can considerably enhance the expression of the target proteins in *N. benthamiana*.

We found that the use of double terminators resulted in an increased fluorescence intensity compared to that of single terminators (Figure S1 and Figure 1), which is consistent with the results of previous studies [17,28,35,36]. The fluorescence intensities of GR were similar to those reported in a previous study [27]; however, those of GS were different. The construct for GFP expression used in the previous study [27] contained a 35S long promoter (1.3 kb), whereas in this study, the 35S dual promoter (0.7 kb; duplicating the enhancer region) drove tGFP expression. The 35S terminator sequences used in the previous study [27] and in this study were similar; however, the reason for using the same 35S terminator and slightly different 35S promoters to express GFP and tGFP as the target proteins, respectively, is unclear and requires further elucidation.

A previous study [35] identified the intensity of terminators by transient GFP expression in *N. benthamiana* in the same manner as the current study; however, ExtT (EU) induced a significantly higher fluorescence than the other terminators, and those of Act3T and PinIIT were similar, which differed from the present results.

The first terminator sequence can alter the activity of the second terminator. 35ST, located upstream of *Agrobacterium* nopaline synthase terminator (NOST), activates a cryptic PA site, changing the dominant PA site in NOST [29]. Furthermore, when GUS expression was tested under the control of the 35S promoter with duplicated enhancers and the NOST, more read-through transcripts were generated because of abnormal transcription termination in the combination containing the NOST than in the combination with the 35ST [28]. The double terminator constructs, which include an additional second terminator, exhibited increased fluorescence intensity compared to the single terminator constructs with only the first terminator (Figure 1b). GRS, GRX (tGFP:RbcST–ExtT), GRP, and GRA (tGFP:RbcST–Act3T), which had terminators added to GR, had the 16th, 11th, 8th, and 5th highest fluorescence intensities, respectively, and all had increased fluorescence intensity compared to that of GR, which had the 17th highest intensity. GAS (tGFP:Act3T–35ST), GAR (tGFP:Act3T–RbcST), and GAX (tGFP:Act3T–ExtT), which had a terminator added to GA had the 13th, 12th, and 10th highest fluorescence intensities, respectively. All had increased fluorescence intensity compared to that of GA, which was ranked 14th. In addition, GPS, GPR, and GPX, which had a terminator added to GP, had the third, second, and first highest fluorescence intensities, respectively; all had increased fluorescence intensity compared to that of GP, which was ranked sixth. These results suggest that the strength of the first terminator determined the strength of the double terminator. A previous study showed that the fluorescence intensity of ExtT was stronger than that of 35ST and that the fluorescence intensity of the ExtT–35ST combination was approximately 1.4-fold higher than that of the 35ST–ExtT combination [35]. The study also tested NOST in combination with 35ST, which had a stronger fluorescence intensity than NOST alone, and the 35ST–NOST combination had 1.6 times the fluorescence intensity of the NOST–35ST combination. Furthermore, the combinations exceeded or were comparable to the fluorescence intensity of ExtT (EU), the best single terminator. These results support the previous assumption that the strength of the first terminator is more crucial than that of the second terminator in double-terminator combinations. However, this result is contradictory when comparing the fluorescence intensities of the reordered combinations of the terminators GRS–GSR, GAR–GRA, GRX–GXR (tGFP:ExtT–RbcST), and GRP–GPR. The fluorescence intensities of GXR and GPR were stronger than those of GRX and GRP, respectively, because ExtT and PinIIT are stronger than RbcST (Figure 1d and Figure S1b). However, because GR and GS have the lowest fluorescence intensity and are challenging to distinguish, it is unlikely that the >2-fold higher fluorescence intensity of GRS compared to that of GSR is caused by 35ST being stronger than RbcST. Furthermore, the fact that GAR is weaker than GRA and that GA shows stronger fluorescence than GR negates this assumption (Figure 1d). Therefore, these results suggest that the strength of the first terminator primarily determines the intensity of the double terminators and is somewhat influenced by the strength of the second terminator. Furthermore, achieving harmonious optimization between the two terminators is also crucial.

The fluorescence intensity of the GS-infiltrated leaf sample was only 10% of that of the control (GO-infiltrated leaf sample), while that of the IR:GS-infiltrated leaf sample increased to 77% of that of the control (Figure 1d and Figure S2b). Two hypotheses can be proposed as to why this may have occurred. First, the IR sequence following 35ST may have helped 35ST restore its original strength. Second, the IR sequence may have hindered the other terminators from maintaining their strength, but it may have specifically enhanced the strength of 35ST. However, how IR affects the strength of the bioparts, such as neighboring terminators or promoters, remains unclear.

Compared with that of the control leaf sample, the fluorescence intensity significantly increased by 4.1-fold in the GPX-infiltrated leaf sample, 3.7-fold in the IR:GS+T–C123-infiltrated leaf sample, and 8.3-fold in the IR:GPX+T–C123-infiltrated leaf sample (Figure 1d,e), showing that the TYLCV-based viral vector system and the use of a double terminator have a synergistic effect on the increase in tGFP fluorescence intensity, respectively. A previous study compared transient tGFP expression by co-infiltration with IR:GP and one of the constructs containing replication-related protein from TYLCV (T–C1, T–C12, and T–C123) [37]. The tGFP fluorescence level of leaves co-infiltrated with T–C123 was approximately 1.2-fold higher than that of leaves co-infiltrated with T–C1 at 3 DPI. In this study, co-infiltration of T–C1 or T–C123 with constructs containing a single or 10 double terminators resulted in slightly higher tGFP fluorescence when co-infiltrated with T–C123 than with T–C1; however, this result was not significant. In contrast, co-infiltration with T–C123 increased the fluorescence for leaf samples infiltrated with IR:GRP, IR:GRA, and IR:GRX, which showed relatively low fluorescence intensity upon co-infiltration with T–C1 (Figure 2d,e). This finding could be due to the fact that co-infiltration with T–C1 did not result in sufficient protein expression for constructs containing relatively weak terminators, but co-infiltration with T–C123 resulted in synergistic protein accumulation. However, for constructs containing relatively strong terminators, co-infiltration with T–C1 seemed to be comparable to tGFP production and co-infiltration with T–C123.

It is widely recognized that co-infiltration of P19 significantly contributes to the suppression of PTGS, thereby increasing the content of the target protein [9,12,37,40]. The target protein accumulates most at 3–5 DPI in transient expression and 7–8 DPI in co-expression with P19 [9,12,40]. However, tGFP expression was highest at 3 DPI when co-expressed with TYLCV Rep and P19 in this study (Figure 3a). This finding is consistent with the reports that transient expression of recombinant proteins using TYLCV-based vectors results in the highest accumulation of target proteins at 3 DPI, owing to the rapid generation of episomal replicons [8,41]. Furthermore, the accumulation of large amounts of heterologous proteins acts as a stressor within plant cells, causing tissue necrosis, and protein accumulation gradually decreases after 3 DPI [31]. In summary, in this study, the fluorescence intensity of tGFP increased when tGFP, TYLCV Rep, and P19 were co-infiltrated, and protein accumulation was highest at 3 DPI and decreased at 5 DPI (Figure 3e,f).

Examples of enhanced recombinant protein expression using geminivirus-based vectors include bean yellow dwarf virus (BeYDV)-based vector, which was used to produce hepatitis B core antigen (0.8 mg/g LFW), Norwalk virus capsid protein (0.34 mg/g LFW) [42], and anti-Ebola monoclonal IgG 6D8 (0.5 mg/g LFW) in *N. benthamiana* leaves as a transient expression system [43]. In addition, GUS expression increased by approximately 10% compared to that of TSP when the INPACT system was used based on the replication mechanism of tobacco yellow dwarf mastrevirus [8]. A high quantity of tGFP was observed in the co-expression with TYLCV C123 (up to 1.14 mg/g LFW), honeysuckle yellow vein virus C123 (up to 1.1 mg/g LFW), and beet mild curly top virus C1 (up to 0.69 mg/g LFW), respectively, which were all co-expressed with P19 [37]. Compared to these studies using PinIIT, tGFP expression was significantly increased by up to 1.47 mg/g LFW in this study using TYLCV Rep and a *tGFP* transcription unit containing PinIIT–ExtT double terminators, showing a 29% increase by only adding ExtT. This result suggests that the expression of target proteins can be increased by including the combination of the high-stringency double terminators identified in various other viral vector systems in this study.

The analysis of PA sites showed that the PA site at the first terminator was less variable, whereas that at the second terminator was more variable than when the terminator was located first. When the first terminator was stronger than the second terminator, the PA site of the second terminator changed significantly compared with when the terminator was located first (Figure 5g,i). Even when the first terminator was weaker than the second terminator, the PA site of the second terminator was slightly altered, with a minor PA site shift compared to the original (Figure 5f,j). These results suggest that the first terminator promotes gene expression by modifying the PA site at the second terminator and that the magnitude of the modification is proportional to the terminator strength. This finding is consistent with the results reported by Sanfaçon et al. [29] that 35ST, located upstream of the NOST, changes the primary PA site of the subsequent NOST.

The tGFP content of the GPR-infiltrated leaf sample was approximately 1.7-fold higher than that of the GRP-infiltrated leaf sample, suggesting that the multiple PA site features of RbcST may influence the structure of *tGFP* mRNA, depending on its position in the double terminator construct. For genes with numerous PA sites, alternative PAs produce different mRNA isoforms with varying gene regulatory abilities and mRNA stability [44,45,46,47,48]. These isoforms—which encode the same protein but have different 3′-UTR lengths—regulate tissue- and developmental stage-specific gene expression and are influenced by physiological conditions such as cell growth, differentiation, and development [39,44,45,46,47]. Longer mRNAs contain more regulatory elements than shorter mRNAs, potentially leading to miRNA degradation and inhibition or promoting translation by RNA-binding proteins [44,45]. Thus, GPRs have fewer alternative PAs and shorter mRNA, whereas GRPs have more alternative PAs and longer mRNAs. These factors may also affect protein accumulation, suggesting that GPRs may result in higher protein content than GRPs.

In the analysis of PA sites, we observed that abnormal transcription was compensated for by combining two terminators, resulting in increased tGFP expression. However, it was difficult to determine the PA rate between each terminator region when two terminators were combined. Therefore, it is necessary to analyze the strength of each terminator and the PA rate at each terminator when these terminators are combined. Furthermore, by analyzing the strength of each terminator and the pattern that compensates for abnormal transcription by combining these terminators, which terminator is selected will be an important consideration in constructing a high-expression vector for the target protein.

In conclusion, the tGFP fluorescence levels were enhanced more in the leaf samples infiltrated using double terminators than using single terminators, with increases of up to 4.1-fold compared to that of the GO-infiltrated sample. Among the single terminators, tGFP fluorescence levels were the highest when PinIIT was used, and PinIIT–ExtT was the highest among the double terminators. When these constructs were transiently co-expressed with TYLCV Rep, the fluorescence intensity increased up to 8.3-fold compared to that of GO and up to 23.7-fold when P19 was transiently co-expressed. The accumulation of tGFP was dramatically enhanced using the TYLCV Rep and P19 in the double terminator. Furthermore, the first terminator influenced the PA site determination of the second terminator in the double-terminator construct. Therefore, the results of this study show that when a double terminator is used, the second terminator complements the transcriptional termination function of the first terminator, which increases the expression of the target protein. Furthermore, the expression of the target protein was increased by increasing the episomal replicon of the target gene using TYLCV Rep and by suppressing PTGS using P19. Therefore, this study has implications in that it can increase the production of the target protein, such as vaccines and pharmaceuticals in plant systems using the double terminators, suppressing PTGS, and combining with previously developed deconstructed TYLCV-based vectors. Furthermore, improved plant expression systems based on the TYLCV-based vector and double terminator will make large-scale production of target protein more viable, which could reduce costs.

## 4. Materials and Methods

### 4.1. Plant Materials and Growth Conditions

*N. benthamiana* plants were grown in commercial potting soil in a greenhouse and using an automated supplied hydroponic system (KAST Engineering, Gumi, Republic of Korea), respectively. The growth conditions in the greenhouse were 24 °C and 16 h light/8 h dark. The conditions in the growth chamber for the hydroponics systems were as follows: 23 °C, 16 h light/8 h dark at a light intensity of 200 mol m^−2^s^−1^, the nutrient solution was adjusted to pH 6.0, and 1.0 electroconductivity was supplied for 1 min once every 5 min. A modified version of the nutrient solution of Yamazaki [49] was used for hydroponic culture (Table S1). All *N. benthamiana* plants used in the experiments were 4–5 weeks old after sowing.

### 4.2. Vector Construction

The 35S dual promoter and TMV Ω 5′-UTR were used to express the target protein *Pontellina plumata* plant codon-optimized tGFP (pICSL80005) using the modular cloning (MoClo) system employing the Golden Gate method. Five single terminators (35ST (pICH41414), RbcST (pICH71411), Act3T, ExtT, and PinIIT) combined with 13 dual terminators were used to build vector constructs (Figure 1a,b). The *Bpi*I recognition site in Act3T was removed using PCR amplification using a modified primer set for Act3T subcloning to the Level 0 vector. ExtT has been used as an intronless extension terminator (EU) [26,35]. The vectors were named using the G of tGFP and the representative letter of each terminator (OCS, O; 35S, S; RbcS, R; Act3, A; Ext, X; and PinII, P). The 35S dual promoter, Ω, tGFP, and a single terminator were inserted into the Level 1 vector, a binary vector with a carbenicillin resistance gene as an *E. coli* selection marker. The *tGFP* transcription unit in the completed Level 1 vector and the second terminator were inserted together into the Level M vector, a binary vector with a spectinomycin resistance gene as an *E. coli* selection marker, to create a vector with a double terminator. Replicating vectors were constructed in the Level P vector, a binary vector with a kanamycin resistance gene as an *E. coli* selection marker, by inserting TYLCV IR to both ends of the *tGFP* transcription unit in Level M vector (Figure 2a). Both for the expression of TYLCV replication initiation protein (Rep, C1) and for the co-expression of TYLCV replication initiation protein (Rep, C1) (C1) + transcriptional activator protein (TrAP, C2) + replication enhancer (REn, C3), C1 and C123 were inserted into the Level 2 vectors, a binary vector containing CaMV 35S short promoter and *A. tumefaciens* NOS terminator with a kanamycin resistance gene as an *E. coli* selection marker, in our previous study (pT–C1 and pT–C123, respectively) [37] (Figure S1a,b). TBSV P19 was used to suppress gene silencing, which was controlled by the 35S promoter and 35S terminator [12,35]. Each binary vector was introduced into the *A. tumefaciens* GV3101 strain via the freeze and thaw method using 1 µg recombinant plasmid [50]. Level 0, 1, M, and P vectors mentioned above included the MoClo tool kit in addgene (#1000000044). The primers used in this study and their terminator sequences are listed in Tables S2 and S3, respectively.

### 4.3. Transient Expression in N. benthamiana Leaves

The *A. tumefaciens* GV3101 strain containing each construct was cultured in 5 mL of Luria–Bertani broth with 50 mg/L rifampicin and the necessary antibiotics for the plasmid vector. The culture was incubated for 1 d in a 28 °C shaking incubator and then centrifuged at 3500× *g* for 5 min, and the pellet was resuspended in infiltration buffer (10 mM 2-(N-morpholino) ethanesulfonic acid, 10 mM MgCl_2_, pH 5.6, 200 μM acetosyringone) to an OD_600_ of 0.8 [11]. Agroinfiltration was performed on the abaxial leaves of 4–5-week-old *N. benthamiana* plants using a 1-mL needleless syringe [11]. *Agrobacterium*-carrying constructs for tGFP expression and replication (pT–C1 or pT–C123) were co-infiltrated at a ratio of 1:1 (*v*/*v*). *Agrobacterium* carrying the constructs for tGFP expression, replication, and P19 were co-infiltrated at a ratio of 2:2:1 (*v*/*v*/*v*). Three plants were used, with two moderately large young leaves at the top of each plant, and the experiment was repeated in triplicate.

### 4.4. tGFP Fluorescence Analysis and Quantification

tGFP fluorescence intensity was measured and compared using a luminescent image analyzer LAS-4000 (Fujifilm, Tokyo, Japan) at 3 and 5 DPI. Subsequently, infiltrated samples from different leaves were pooled and pulverized using a TissueLyser II (QIAGEN, Hilden, Germany), and tGFP fluorescence was quantified at a wavelength of 485/535 nm (excitation/emission) using a GFP quantification kit (Abcam, Cambridge, UK) and a microplate reader. The tGFP quantification value for each sample was calculated using a tGFP standard calibration curve. The tGFP quantification results of each 3 DPI sample were subjected to one-way analysis of variance (ANOVA) at the 95% confidence level and post hoc analysis using Tukey’s test (*p* < 0.05). Wilcoxon’s signed-rank test was performed at the 95% confidence level to determine whether there was a significant difference between the tGFP quantification results of the 3 and 5 DPI samples.

### 4.5. Protein Extraction and SDS-PAGE

TSPs were extracted by adding 150 μL of extraction buffer (50 mM Tris-Cl (pH 7.4), 250 mM sucrose, 50 mM NaCl, 0.2% Triton X-100, 10% glycerol, 5 mM 1,4-dithiothreitol, and 1 mM phenylmethylsulfonyl fluoride) to 50 mg of pulverized infiltrated sample. TSPs were quantified using a DC protein assay kit (BIO-RAD, Hercules, CA, USA), and 25 μg of TSP was loaded onto a manually prepared 10% SDS-PAGE gel and electrophoresed. As a positive control, 100 ng of tGFP standard (Evrogen, Moscow, Russia) was loaded, and 25 μg of TSP from the infiltrated sample with an empty vector was loaded as a negative control. After electrophoresis, the sections were stained with Coomassie Brilliant Blue staining solution (MeOH:H_2_O:acetic acid = 45:45:10, 0.1% Coomassie Brilliant Blue R-250) for 1 h and then destained with destaining solution (MeOH:H_2_O:acetic acid = 45:45:10) for 2 h at 25 °C.

### 4.6. ELISA

TSP from the infiltrated sample was diluted appropriately and used for indirect ELISA. Recombinant tGFP standards, samples, and blanks were coated on immunoplates (Thermo Fisher Scientific, Waltham, MA, USA) in serial dilutions with coating buffer (0.1 M NaHCO_3_, 0.03 M Na_2_CO_3_). The immunoplate was then blocked with 1% bovine serum albumin (in 1× phosphate-buffered saline) solution. For tGFP-antibody binding, anti-tGFP mouse IgG (1:2000, OriGene, Rockville, MD, USA) was used as the primary antibody, and horseradish peroxidase-conjugated anti-mouse goat IgG (1:10,000, Invitrogen, Carlsbad, CA, USA) was used as the secondary antibody. To detect the signal, BD OptEIA^TM^ 3,3′,5,5′-tetramethylbenzidine substrate (BD Biosciences, Franklin Lakes, NJ, USA) was added and incubated for 20 min at room temperature. Color development was determined at a wavelength of 450 nm after adding a stopping solution (0.2 M H_3_PO_4_). The quantitative value for each sample was calculated using a recombinant tGFP standard curve.

### 4.7. cRT-PCR for Analyzing the PA Sites

cRT-PCR was performed as previously described [26]. Total RNA was extracted from each infiltrated sample using the Spectrum^TM^ Plant Total RNA Kit (Sigma-Aldrich, St. Louis, MO, USA). Total RNA (5 µg) was reacted with 25 U of RNA 5′-pyrophosphohydrolase (New England Biolabs, Ipswich, MA, USA), 40 U of RNAseOUT recombinant ribonuclease inhibitor (Invitrogen), and ThermoPol reaction buffer (New England Biolabs) to a final volume of 50 μL, reacted at 37 °C for 1 h to remove the 5′-cap, and inactivated at 65 °C for 5 min by adding 1 μL of EDTA. Decapped RNA was purified using the Spectrum^TM^ Plant Total RNA Kit, using 10 U of T4 RNA ligase 1 (New England Biolabs), T4 RNA ligase reaction buffer (New England Biolabs), 50 μM ATP, 10% PEG, and 40 U RNAseOUT recombinant ribonuclease inhibitor (Invitrogen) in a final volume of 20 μL, circulated at 25 °C for 90 min, and inactivated at 100 °C for 2 min. First-strand cDNA was synthesized using tGFP 5′ reverse primer and the PrimeScript 1st strand cDNA Synthesis kit (Takara, Shiga, Japan). The region containing the poly(A) tail was amplified using the tGFP 3′ forward and tGFP 5′ reverse primers. PCR products were purified using a QIAquick PCR Purification Kit (QIAGEN). The primer and terminator sequences used in this study are listed in Tables S2 and S3, respectively.

### 4.8. Polyadenylation Site Analysis Using Next-Generation Sequencing

Purified cRT-PCR products were used as templates for primary PCR amplification, followed by secondary PCR with adaptors at a 10–50-fold dilution. Large PCR products were removed using HiAccuBead (ACCUGENE, Incheon, Republic of Korea). A third round of indexed PCR was performed at 10–50-fold dilution, and the amplified products were pooled to equal concentrations and purified using HiAccuBead to create libraries. Each library was sequenced using the iSeq 100 system (Illumina, San Diego, CA, USA), and paired-end reads from each sample were merged using the FLASH program [51]. Reads with >10 bp of contiguous PA signal sequences within the merged reads were selected and used for further analysis. The 10 bp sequence immediately preceding the PA site in the read was targeted to determine the frequency of reads with that sequence.

## Figures and Tables

**Table 1:** Analysis of putative signal motifs in each construct with a single or double terminator. Because the putative NUE motifs UAAUAA and AAUAAU in RbcST overlapped, it was difficult to determine which NUE motif determined the PA site. All 10 bp sequences before the polyadenylation site had a read rate of >5% in the polyadenylation site analysis results, and each putative far and near upstream element motif was marked.

Construct	Terminator	Putative FUE Motif	Putative NUE Motif	Ranking in Arabidopsis Gene NUEs [38]	10 nt Sequence Before the Polyadenylation Site	Read Rate (%)
GO	OCS	UUGUA	AUGAAU	39	CGTTCAATTT	75.06
			UAAAAU	20	TATTGTGCTG	5.66
GP	PinII	AUGUA GUGUA	GAAUAA	32	TTCTTATCCT	69.06
					GATCATCCAT	12.27
					CATATTTCTT	5.22
GPR	PinII	AUGUA GUGUA	GAAUAA	32	TTCTTATCCT	66.79
					GATCATCCAT	15.04
					CATATTTCTT	6.09
	RbcS	AUGUA	UAAUAA or AAUAAU	3 9	TTGTTTCTTT	35.19
					TAATAATAGT	27.25
					TTTCTTTACT	18.15
					CATTTGTTTC	17.53
e	RbcS	AUGUA	UAAUAA or AAUAAU	3 9	TTTCTTTACT	33.80
					TTGTTTCTTT	28.63
					TAATAATAGT	8.11
					TAATAGTAAT	7.87
					CATTTGTTTC	7.72
	Act3	UUGUA	AUGAAA	29	AGTTCATGTT	28.81
			AUAUAU	7	TTGTCAGTTC	24.95
			UAUAUA	8	CTTGTCAGTT	12.50
					GTTGTATGTG	8.35
			AUUUUU	72	TTTTGGTATC	7.46
GRP	RbcS	AUGUA	UAAUAA or AAUAAU	3 9	TTTCTTTACT	34.21
					TTGTTTCTTT	29.65
					CATTTGTTTC	7.71
					TAATAATAGT	7.01
					TAATAGTAAT	6.72
	PinII	AUGUA	GAAUAA	32	TTCTTATCCT	79.60
		GUGUA	AAUGAA	21	TTCTTTGATG	15.32

## Data Availability

The original contributions presented in the study are included in the article/Supplementary Material, further inquiries can be directed to the corresponding author.

## Supplementary Materials

The following supporting information can be downloaded at https://www.mdpi.com/article/10.3390/ijms252111573/s1.
