# Supplementary material for: High-Level Production of a Recombinant Protein in Nicotiana benthamiana Leaves Through Transient Expression Using a Double Terminator
Source: Int J Mol Sci. 2024 Oct 28;25(21):11573. doi: 10.3390/ijms252111573 (PMC11547012; doi:10.3390/ijms252111573)
Supplement: Supplementary file 1 [file ijms-25-11573-s001.zip › Caption.pdf]

Table S1: Composition of the nutrient solution for hydroponics culture used in this study.

Table S2: List of primers used in this study.

Table S3: Terminator sequences used in this study.

Figure S1: Relative turbo green fluorescent protein (tGFP) fluorescence intensities in greenhouse- and hydroponically-grown *Nicotiana benthamiana* leaf samples agroinfiltrated with the vector constructs for tGFP expression containing single or double terminators. (a) GFP fluorescence of each construct on transient expression in greenhouse-grown *N. benthamiana* at 3 DPI. Scale bar: 1 cm. (b) The relative GFP fluorescence intensity of each construct was calculated using GFP quantitative data quantified using the GFP quantification kit. The results were statistically analyzed using a one-way ANOVA followed by Tukey's test ($P < 0.05$). (c) GFP quantity (μg/g LFW) of each construct upon transient expression in greenhouse-grown and hydroponically grown *N. benthamiana* at 3 DPI was calculated using a GFP quantification kit (nd, not detected). The results were statistically analyzed using Wilcoxon t-test ($P < 0.05$).

Figure S2: Relative tGFP fluorescence intensities in *N. benthamiana* leaf samples agroinfiltrated with vector constructs for tGFP containing double terminators and intergenic regions at both ends. (a) tGFP fluorescence of each construct upon transient expression at 3 DPI. Scale bar: 1 cm. (b) The relative GFP quantity of each construct was calculated using GFP data quantified using the GFP quantification kit. The results were statistically analyzed using a one-way ANOVA followed by Tukey's test ($P < 0.05$).
