# Supplementary material for: High-Level Production of a Recombinant Protein in Nicotiana benthamiana Leaves Through Transient Expression Using a Double Terminator
Source: Int J Mol Sci. 2024 Oct 28;25(21):11573. doi: 10.3390/ijms252111573 (PMC11547012; doi:10.3390/ijms252111573)
Supplement: Supplementary file 1 [file ijms-25-11573-s001.zip › Table S1.pdf]

Table S1. Composition of the nutrient solution for hydroponics culture used in this study. Nutrient solutions A and B were prepared separately at 100X concentration, diluted and used for hydroponic cultivation of *N. benthamiana*.

Nutrient stock	Composition	100X Concentration (mg/L)
Nutrient A	$Ca(NO_3)_2$*4H_2O	6,000
	KNO_3	20,000
	NaFe-EDTA	1,200
Nutrient B	$MgSO_4$*7H_2O	3,690
	H3BO3	150
	$MnSO_4$*5H_2O	100
	$CuSO_4$	2.5
	$NaMoO_4$*2H_2O	1
	$ZnSO_4$*7H_2O	11
	NH_4NO_3	2,000
	KH_2PO_4	3,375
