# Supplementary material for: High-Level Production of a Recombinant Protein in Nicotiana benthamiana Leaves Through Transient Expression Using a Double Terminator
Source: Int J Mol Sci. 2024 Oct 28;25(21):11573. doi: 10.3390/ijms252111573 (PMC11547012; doi:10.3390/ijms252111573)
Supplement: Supplementary file 1 [file ijms-25-11573-s001.zip › Table S2.pdf]

Supplementary Table S1. Primer list used in this study.

Application	Primer name	Primer sequence (5' to 3')
For removal of *Bsa*I enzyme site	NbAct3T w/o typeIIS-F2	TGTCAGTTCATTTTGGTATCATTTT
	NbAct3T w/o typeIIS-R2	GGATACCAAAAAAATGATACCAAAAT
For cloning of Level 0-3UT	*Bpi*I GCTT NtExtT-F	TTGAAGACAAGCTTAAAGCAGAATGCTGAGCTAAAAGAAAG
	*Bpi*I CGCT NtExtT-R	TTGAAGACAAAGCGGTCATAACTGTAGAAATGATTCCATTAC
	*Bpi*I GCTT PinIIT-F	TTGAAGACAAGCTTACCCTGCAATGTGAC
	*Bpi*I CGCT PinIIT-R	TTGAAGACAAAGCGCTGCAGGTCGATTCATAGAAGATTA
	*Bpi*I GCTT NbAct3T-F	TTGAAGACAAGCTTTTACAGCATTCCCAGAAAGAGAAAC
	*Bpi*I CGCT NbAct3T-R	TTGAAGACAAAGCGATGCTAGCTTGTTTACACCTCGATT
For sequencing of Level 0 construct	Lev0 seq-F	CCTGTCGGGTTTCGCCACCT
	Lev0 seq-R	GCCGTTACCACCGCTGCGTT
For cloning of Level 1-3 terminator	*Bsa*I GGAG 35ST-F	TTGGTCTCAGGAGCTCTAGCTAGAGTCGATCGACA
	*Bsa*I CGCT 35ST-R	TTGGTCTCAAGCGATCTGGATTTTAGTACTGGATTTTGGTT
	*Bsa*I GGAG NtExtT-F	TTGGTCTCAGGAGAAAGCAGAATGCTGAGCTAAAAGAAAG
	*Bsa*I CGCT NtExtT-R	TTGGTCTCAAGCGGTCATAACTGTAGAAATGATTCCATTAC
	*Bsa*I GGAG PinIIT-F	TTGGTCTCAGGAGACCCTGCAATGTGAC
	*Bsa*I CGCT PinIIT-R	TTGGTCTCAAGCGCTGCAGGTCGATTCATAGAAGATTA
	*Bsa*I GGAG NbAct3T-F	TTGGTCTCAGGAGTTACAGCATTCCCAGAAAGAGAAAC
	*Bsa*I CGCT NbAct3T-R	TTGGTCTCAAGCGATGCTAGCTTGTTTACACCTCGATT
	*Bsa*I GGAG SlRbcS3CT-F	TTGGTCTCAGGAGATATGTCAACAGTGAGAAACTGTTC
	*Bsa*I CGCT SlRbcS3CT-R	TTGGTCTCAAGCGTTATATAGCAGCCACAAGACTTTC
For sequencing of Level 1 construct	35SP 3'-F	AATCCCACTATCCTTCGCAA
	Lev1 RB-R	GCTCTTTTCTCTTAGGTTTAC
For cRT-PCR	tGFP 3'-F	GTACCAACATGCTTTCAAGACC
	tGFP 5'-R	TCCATAGCAGGAAGTCCAGAC
For analysis of Poly(A) site	OCST FW	TATGAATAATATTCTCCGTTC
	GP FW-1	TTATCTGAATAAGAGAAAGAGATC
	GR FW-1	TTCCAGTTGAATTTATTATGAGAAC
	GA FW-1	TTTCATTTTGAGAGCAGCACC
	GA FW-2	ATATGCTTCTCATTTGTATTTCC
	GA FW-3	TTCTATCCCTTTGTTTGAGTC
	GA FW-4	CAAATATTCGGTCATTGTTGG
	RV	TTGTAATGTTGTTTGTTGTTTGTTG
	Oligo dT	TTTTTTTTTTTTTTTTTT
	OCST adapF	ACACTCTTTCCCTACACGACGCTCTTCCGATCTTATGAATAATATTCTCCGTTC
	GP adapF	ACACTCTTTCCCTACACGACGCTCTTCCGATCTCCCTGCAATGTGACCCTAGA
	GR adapF	ACACTCTTTCCCTACACGACGCTCTTCCGATCTTTCCAGTTGAATTTATTATGAGAAC
	GA-1 adapF	ACACTCTTTCCCTACACGACGCTCTTCCGATCTTTTCATTTTGAGAGCAGCACC
	GA-2 adapF	ACACTCTTTCCCTACACGACGCTCTTCCGATCTATATGCTTCTCATTTGTATTTCC
	GA-3 adapF	ACACTCTTTCCCTACACGACGCTCTTCCGATCTTTCTATCCCTTTGTTTGAGTC
	GA-4 adapF	ACACTCTTTCCCTACACGACGCTCTTCCGATCTCAAATATTCGGTCATTGTTGG
	RV adapR	GTGACTGGAGTTCAGACGTGTGCTCTTCCGATCTTTGTAATGTTGTTTGTTGTTTGTTG
	Oligo dT adapR	GTGACTGGAGTTCAGACGTGTGCTCTTCCGATCTTTTTTTTTTTTTTTTTTT
