# Supplementary material for: High-Level Production of a Recombinant Protein in Nicotiana benthamiana Leaves Through Transient Expression Using a Double Terminator
Source: Int J Mol Sci. 2024 Oct 28;25(21):11573. doi: 10.3390/ijms252111573 (PMC11547012; doi:10.3390/ijms252111573)
Supplement: Supplementary file 1 [file ijms-25-11573-s001.zip › Table S3.pdf]

Supplementary Table S2. Terminator sequences used in this study.

Terminator (Source)	Sequence (length)
35S (Cauliflower mosaic virus; CaMV)	CTCTAGCTAGAGTCGATCGACAAGCTCGAGTTTCTCCATAATAATGTGTGAGTAGTTCCCAG<br>ATAAGGGAATTAGGGTTCCTATAGGGTTTCGCTCATGTGTTGAGCATATAAGAAACCCTTAG<br>TATGTATTTGTATTTGTAAAATACTTCTATCAATAAAATTTCTAATTCCTAAAACCAAAATCCA<br>GTACTAAAATCCAGAT (204 bp)
RbcS3C (*Solanum lycopersicum*)	ATATGTCAACAGTGAGAAACTGTTCGCATTTTCCGTTTTGCTTCTTTCTTTCTATTCAATGTA<br>TGTTGTTGGATTCCAGTTGAATTTATTATGAGAACTAATAATAATAGTAATAATCATTTGTTTC<br>TTTACTAATTTGCATTTTCACATATGATTTCTGGTGCATATCATAATTTTCATTCCACCAATATT<br>AATTTCCCCCATTCAAGTTACTTATGAAATAGAAATCCTCTTCTCCGACTACTTTATTTGTCC<br>GAAAGTCTTGTGGCTGCTATATAA (279 bp)
PinII (*Solanum tuberosum*)	ACCCTGCAATGTGACCCTAGACTTGTCCATCTTCTGGATTGGCCAACTTAATTAATGTATGA<br>AATAAAAGGATGCACACATAGTGACATGCTAATCACTATAATGTGGGCATCAAAGTTGTGTG<br>TTATGTGTAATTACTAATTATCTGAATAAGAGAAAGAGATCATCCATATTTCTTATCCTAAATG<br>AATGTCACGTGTCTTTATAATTCTTTGATGAACCAGATGCATTTTATTAACCAATTCCATATAC<br>ATATAAATATTAATCATATATAATTAATATCAATTGGGTTAGCAAAACAAATCTAGTCTAGGTG<br>TGTTTTGCTAATTATTGGGGGATAGTGCAAAAAGAAATCTACGTTCTCAATAATTCAGATAG<br>AAAACTTAATAAAGTGAGATAATTTACATAGATTGCTTTTATCCTTTGATATATGTGAAACCA<br>TGCATGATATAAGGAAAATAGATAGAGAAATAATTTTTTACATCGTTGAATATGTAAACAATT<br>TAATTCAAGAAGCTAGGAATATAAATATTGAGGAGTTTATGATTATTATTATTATTTTGATGTT<br>CAATGAAGTTTTTTTTAATTTCATATGAAGTATACAAAAATTCTTCATAGATTTTTGTTTCTAT<br>GCCGTAGTTATCTTTAATATATTTGTGGTTGAAGAAATTTATTGCTAGAAACGAATGGATTGT<br>CAATTTTTTTTTAAAGCAAATATATATGAAATTATACTGTATATTATTTTAGTCATGATTAAAAT<br>GTGGCCTTAATTGAATCATCTTTCTCATTCATTTTTTTCAAAAGCATATCAGGATGATTGATATT<br>TATCTATTTTAAAAATTAATTTAAGGGTTCAAATTAAATTTAACTTAAAAGTGTCCTAACCGT<br>AGTTAAAGGTTTACTTTAAAAAAATACTATGAAAAATCTAATCTTCTATGAATCGACCTGCA<br>G (950 bp)
Extensin (*Nicotiana tabacum*)	AAAGCAGAATGCTGAGCTAAAAGAAAGGCTTTTTCCATTTTCGAGAGACAATGAGAAAAG<br>AAGAAGAAGAAGAAGAAGAAGAAGAAGAAGAAGAAGAAAAGAGTAAATAATAAAGCCC<br>CACAGGAGGCGAAGTTCTTGTAGCTCCATGTTATCTAAGTTATTGATATTGTTTGCCCTATAT<br>TTTATTTCTGTCATTGTGTATGTTTTGTTCAGTTTCGATCTCCTTGCAAAATGCAGAGATTAT<br>GAGATGAATAAACTAAGTTATATTATTATACGTGTTAATATTCTCCTCCTCTCTCTAGCTAGCC<br>TTTTGTTTTCTCTTTTTCTTATTTGATTTTCTTTAAATCAATCCATTTTAGGAGAGGGCCAGG<br>GAGTGATCCAGCAAAACATGAAGATTAGAAGAAACTTCCCTCTTTTTTTTCCTGAAAACAA<br>TTTAACGTCGAGATTTATCTCTTTTTGTAATGGAATCATTTCTACAGTTATGAC (486 bp)
Actin3 (*Nicotiana benthamiana*)	TTACAGCATTCCCAGAAAGAGAAACAGAAGAAATATACAAACTTTCATTTTGAGAGCAGC<br>ACCTCGTCTATTGATTGCAGATAATATGCTTCTCATTTGTATTTCCTTTTGATTATTTTTGTTTC<br>TATCCCTTTGTTTGAGTCAATCTCAAATATTCGGTCATTGTTGGTATGAAAAATCAAGCAGTT<br>CATGTTAAGAGTCAATTTAAAATTAATATTTTTATATAGAGTTGTATGTGAAATGATGTTGTG<br>ATTTGGTATATATGGATAAAGAGCTTGTCAGTTCATTTTGGTATCATTTTTTTGGTATCCAAAT<br>AAGAAACACAAAAGGGATATGTCCCTCTACTATCAAATATTAGTTATAAGTATTCATGTTATA<br>CTATTCGATATTTTCTACCCCAATCGTTACCTATTTAAAAGTATTTACCCCTCCATCTATCAAA<br>CCCCTGGACCCAGCTTTCCTATTACATGTGGCTTCATCTTAAGCCCCCAAACCTTTTTCTTAT<br>TTTTGATTTTTAAAGGCTCATCTTAAAATTTATTACTCAAATTAATACCTCTTAATAACCCACC<br>TCAAGGACCCAGTAATTAAATATCCAATTAGCTCCAGTAATTGGGGTTCATATTAGCTCCAG<br>TCTTAAATTTTAAAGGCGATGATCGTATTCCTCCACTTGGTTCATTTATACTCAAAGAATACT<br>CAATGTCTTTAGTGTTTAGATAACTTTTTGTAAATCATATAGATTGTTTTAACAAAAAACAAT<br>TCAATAGTAGATTTTCACATGAAAGTTACATAAAAATTCTTTAAAATTACTTTCTCAAAAAAT<br>TGTTCCAAACATATTATCCCACAATTAAACTCAATCTGTTTTTCGAAACCTAAATCAAAACC<br>AATCCAACTACCTTATATAATATATAATCAATACATTGTAAAGAACTGCATGTTCTTTTAAATT<br>TTGGGGGCAAAGTTATTCCGTACGTTCACACATGTACTAATAGGAGGTAATAAATGATATGT<br>GAAACAATCGAGGTGTAAACAAGCTAGCAT (1038 bp)
