# Supplementary figures and images for: High-Level Production of a Recombinant Protein in Nicotiana benthamiana Leaves Through Transient Expression Using a Double Terminator
Source: Int J Mol Sci. 2024 Oct 28;25(21):11573. doi: 10.3390/ijms252111573 (PMC11547012; doi:10.3390/ijms252111573)

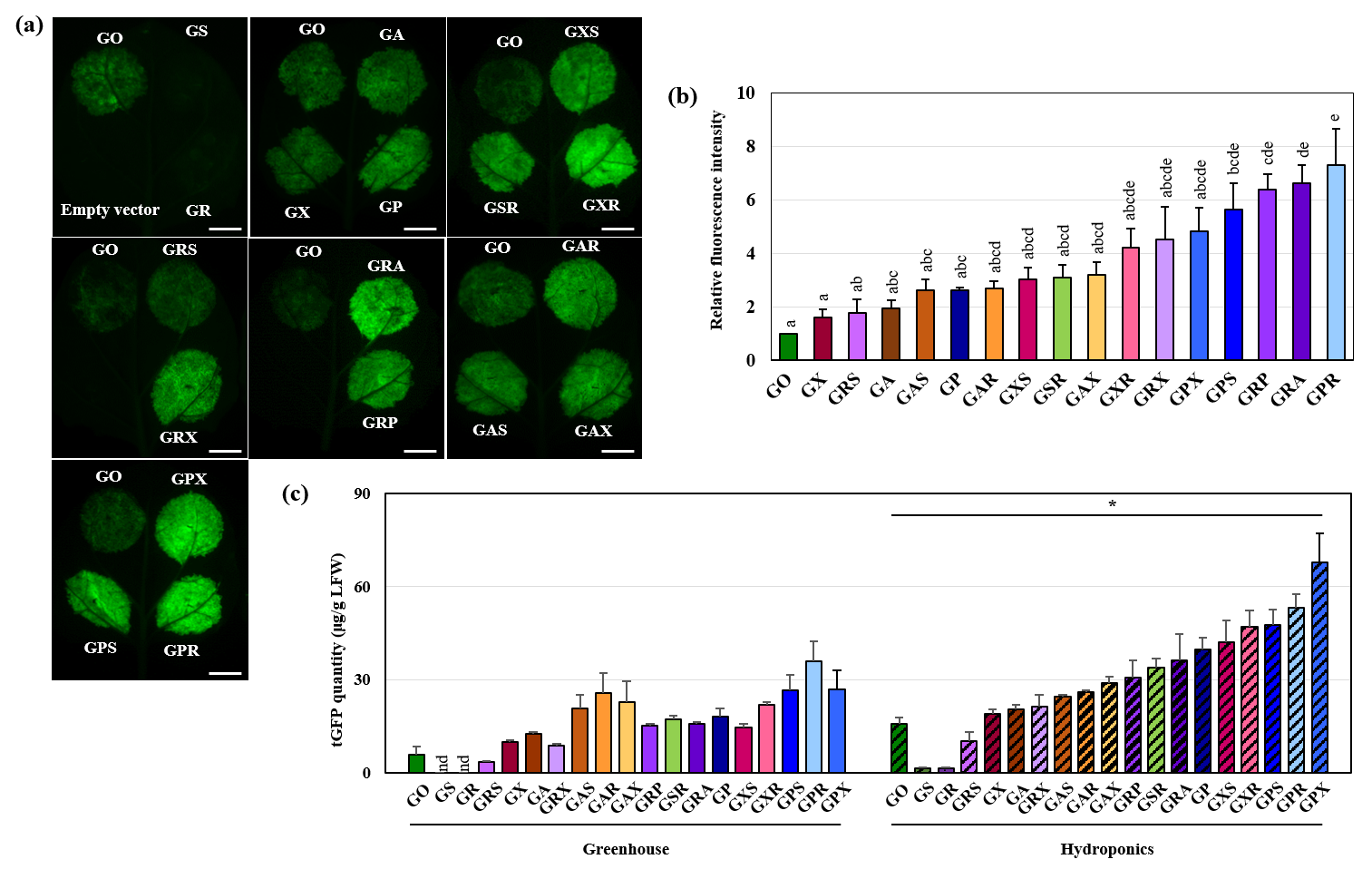

Supplement: Supplementary file 1 [file ijms-25-11573-s001.zip › Fig.S1.tif]

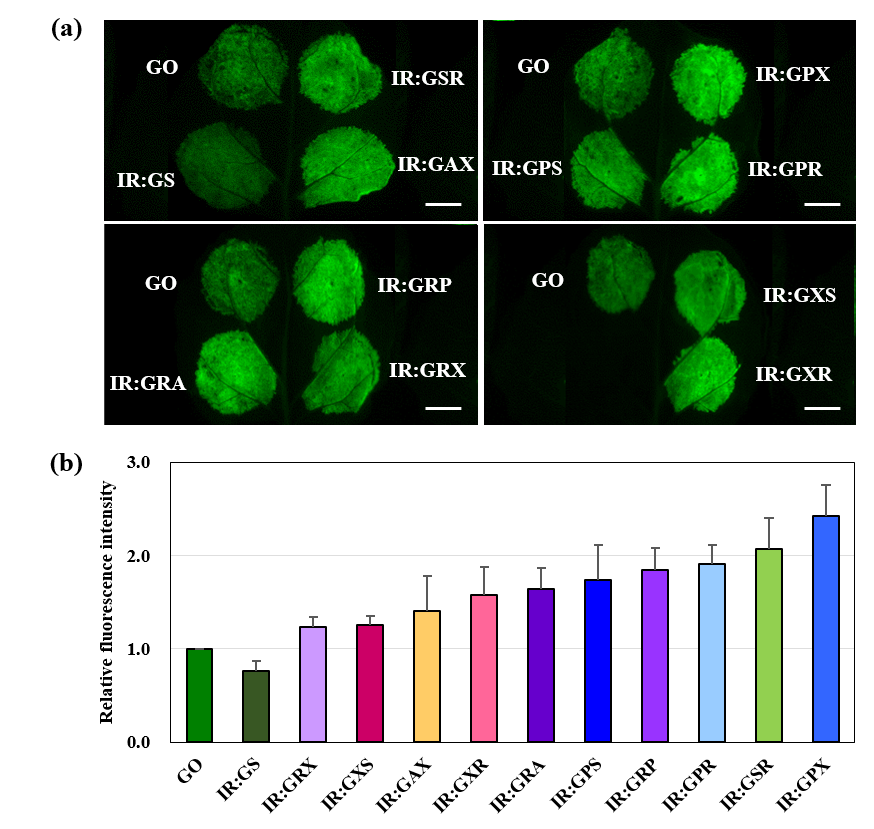

Supplement: Supplementary file 1 [file ijms-25-11573-s001.zip › Fig.S2.tif]
